# Principal component approach in variance component estimation for international sire evaluation

**DOI:** 10.1186/1297-9686-43-21

**Published:** 2011-05-24

**Authors:** Anna-Maria Tyrisevä, Karin Meyer, W Freddy Fikse, Vincent Ducrocq, Jette Jakobsen, Martin H Lidauer, Esa A Mäntysaari

**Affiliations:** 1 Biotechnology and Food Research, Biometrical Genetics, MTT Agrifood Research Finland, 31600 Jokioinen, Finland; 2 Animal Genetics and Breeding Unit, University of New England, Armidale NSW 2351, Australia; 3 Department of Animal Breeding and Genetics, SLU, Box 7023, S-75007 Uppsala, Sweden; 4 UMR 1313 INRA, Génétique Animale et Biologie Intégrative, 78352 Jouy-en-Josas Cedex, France; 5 Interbull Centre, Department of Animal Breeding and Genetics, SLU, Box 7023, S-75007 Uppsala, Sweden

## Abstract

**Background:**

The dairy cattle breeding industry is a highly globalized business, which needs internationally comparable and reliable breeding values of sires. The international Bull Evaluation Service, Interbull, was established in 1983 to respond to this need. Currently, Interbull performs multiple-trait across country evaluations (MACE) for several traits and breeds in dairy cattle and provides international breeding values to its member countries. Estimating parameters for MACE is challenging since the structure of datasets and conventional use of multiple-trait models easily result in over-parameterized genetic covariance matrices. The number of parameters to be estimated can be reduced by taking into account only the leading principal components of the traits considered. For MACE, this is readily implemented in a random regression model.

**Methods:**

This article compares two principal component approaches to estimate variance components for MACE using real datasets. The methods tested were a REML approach that directly estimates the genetic principal components (direct PC) and the so-called bottom-up REML approach (bottom-up PC), in which traits are sequentially added to the analysis and the statistically significant genetic principal components are retained. Furthermore, this article evaluates the utility of the bottom-up PC approach to determine the appropriate rank of the (co)variance matrix.

**Results:**

Our study demonstrates the usefulness of both approaches and shows that they can be applied to large multi-country models considering all concerned countries simultaneously. These strategies can thus replace the current practice of estimating the covariance components required through a series of analyses involving selected subsets of traits. Our results support the importance of using the appropriate rank in the genetic (co)variance matrix. Using too low a rank resulted in biased parameter estimates, whereas too high a rank did not result in bias, but increased standard errors of the estimates and notably the computing time.

**Conclusions:**

In terms of estimation's accuracy, both principal component approaches performed equally well and permitted the use of more parsimonious models through random regression MACE. The advantage of the bottom-up PC approach is that it does not need any previous knowledge on the rank. However, with a predetermined rank, the direct PC approach needs less computing time than the bottom-up PC.

## Background

Globalization of dairy cattle breeding requires accurate and comparable international breeding values for dairy bulls. The international Bull Evaluation Service, Interbull, has for years performed international genetic evaluations for dairy cattle for several traits, serving the cattle breeders worldwide. Due to different trait definitions and evaluation models in countries participating in the international genetic evaluation of dairy bulls, biological traits like protein yield are treated as different, but genetically correlated traits across countries [1]. Therefore, each bull will have a breeding value on the base and scale of each participating country. For protein yield in Holstein, this currently leads to 28 breeding values per bull and the number of partipating countries is expected to increase. Such a model is challenging for those responsible for the evaluations and estimation of the corresponding genetic parameters. The size of the (co)variance matrix is large: for 28 traits, the genetic covariance matrix of the classical, unstructured, multiple-trait model comprises 406 distinct covariance components. Furthermore, the full rank model becomes over-parameterized due to high genetic correlations. In addition, links between populations are determined by the amount of exchange of genetic material among the populations and can vary in strength. These special characteristics have led to a situation, where variance components e.g. for protein yield in Holstein are estimated in sub-sets of countries, and are then combined to build-up a complete (co)variance matrix [2,3]. Also, country sub-setting is not problem-free since it is often necessary to apply a "bending" procedure in order to obtain a positive definite (co)variance matrix when combining estimates from the analyses of sub-sets [4]. Even if the complete data could be analyzed simultaneously, variance component estimation would remain a challenge since the usual estimation methods are very slow or unstable, when the (co)variance matrices are ill-conditioned. Mäntysaari [5] has hypothesized that with the high genetic correlations among countries, estimation of parameters for the full size (co)variance matrix may underestimate the genetic correlations and yield unexpected partial correlations. As an extreme case, this can result in a situation where the bull's daughter performance in one country can effect negatively the bull's EBV in another country. This has been illustrated by van der Beek [6].

Different solutions have been proposed to deal with the problem of over-parameterisation. Madsen et al. [7] have introduced a modification of the average information (AI) algorithm that could be applied to estimate heterogeneous residual variance, residual covariance structure and matrices of reduced rank. Rekaya et al. [8] have employed structural models to estimate genetic (co)variances. They modelled genetic, management and environmental similarities to explain the genetic (co)variance structure among countries and to obtain more accurate estimates of genetic correlations. The authors considered the method useful, especially when there was a lack of genetic ties between countries. However, they noted a 15 to 20% increase in computing time compared to the standard multivariate model. Leclerc et al. [9] have approached the structural models in a different way. They selected a subset of well-connected base countries to build a multi-dimensional space. The coordinates defined by these countries were used to estimate a distance between base countries and other countries and thus the genetic correlations between them. This decreased the number of parameters to be estimated compared to the unstructured variance component matrix for the multiple-trait across country evaluation (MACE) approach [10]. However, when they studied a field dataset, a relatively large number of dimensions was needed to model the genetic correlations appropriately and the estimation process often led to local maxima, decreasing the utility of the approach.

The principal component (PC) approach has also been investigated as a possible solution to deal with the problems of variance component estimation for the international genetic evaluation of dairy bulls. This approach is of special interest because it allows for a dimension reduction. Principal components are independent, linear functions of the original traits. PC are obtained through an eigenvalue decomposition of a covariance or correlation matrix, which yields its eigenvectors and corresponding eigenvalues. Eigenvalues describe the magnitude of the variance that the eigenvectors explain. For highly correlated traits, the first few principal components explain the major part of the variation in the data and those with the smallest contribution on the variance can be excluded without notably altering the accuracy of the estimates, e.g. [11]. Factor analysis (FA) is closely related to the PC approach, but it models part of the variance to be trait-specific. Thus, generally it does not lead to a reduction in rank (assuming all trait-specific variances are non-zero), but benefits from the more parsimonous structure of the (co)variance matrix. Leclerc et al. [12] have studied both PC and FA approaches, but instead of estimating parameters directly from the complete data, they used a subset of well-linked base countries, performed a dimension reduction for the subset and estimated a contribution of the other countries to these PC or factors.

The above studies were motivated by an attempt to reduce the number of parameters in the variance component estimation for MACE, but except for the study of Rekaya et al. [8], they were based on data sub-setting. Kirkpatrick and Meyer [13] and Mäntysaari [5] have suggested two different PC approaches meant to use complete datasets. Kirkpatrick and Meyer [13] have introduced a direct PC approach that exploits only leading principal components to model the variation in a multivariate system to improve the precision of the estimation and to reduce the computational burden inherent in the analysis of large and complex datasets. However, the approach was not specifically designed for MACE and has not been tested for such datasets. The bottom-up PC approach, introduced by Mäntysaari [5], is based on the random regression (RR) MACE model that enables rank reduction. It adds traits, i.e. countries, sequentially in the analysis and defines a correct rank in each step, until all countries are included and the final rank is determined. The bottom-up PC approach was designed to estimate the genetic parameters of large, over-parameterized datasets, for which the estimation of the complete, full rank dataset might not be possible. So far it has only been tested on a simulated dataset. This article studies the value of the direct and the bottom-up PC approaches to estimate the variance components for MACE using real datasets and evaluates the validity of the bottom-up PC approach to determine the appropriate rank of the (co)variance matrix.

## Methods

### Random regression MACE

Classical MACE [10] including *t *countries is applied using the model(1)

where **y***_i _*is a *n_i _*vector of national de-regressed breeding values for bull *i*, **b **is a vector of *t *country effects, **u***_i _*is a vector of *t *different international breeding values for bull *i *and *ε_i _*is a *n_i _*vector of residuals. **X***_i _*and **Z***_i _*are incidence matrices and the variance of the bull's breeding values is Var(**u***_i_*) = **G**. Differences in residual variances, var(*ε_i_*), were taken into account by carrying out a weighted analysis. Specifically, this involved fitting residual variances at unity and scaling the other terms in the model (1) with weights, *w_ij _*= *EDC_ij_/g_jj_λ_j_*, where *g_jj _*is the sire variance of the j'th country,  with heritabilities  provided by each participating country *j *and *EDC_ij _*is the bull's effective daughter contribution in country *j *[14]. Contrary to the official MACE evaluations, in this study animals with unknown parentage were not grouped into phantom parent groups.

Following [5], the genetic (co)variance matrix of the sire effects can be rewritten as(2)

and **C **can be further decomposed into(3)

in which **S **is a diagonal matrix of genetic standard deviations, **C **is a genetic correlation matrix, **D **is the matrix of eigenvalues of **C **and **V **is the matrix of the corresponding eigenvectors. This allows the classical MACE model to be rewritten as an equivalent random regression MACE model [5,15]:(4)

where ***ν***_*i *_is a vector of *t *regression coefficients for bull *i *with var(***ν***_*i*_) = **D**.

### Estimation of the G matrix with appropriate rank

Formulating the classical MACE model as a RR MACE model enables a rank reduction of the genetic (co)variance matrix [16]. If **G **is close to singular, then the *r *largest eigenvalues, *r < t*, explain the essential part of the variance in **G**. Thus, **G **can be replaced with(5)

where the *r *× *r ***D***_r _*contains the *r *largest eigenvalues and the *t × r *matrix **V***_r _*the *r *corresponding eigenvectors [17]. Consequently, *t × t *matrix **G***_r _*has now only *r*(2*t *- *r *+ 1)/2 parameters.

### Bottom-up PC approach

The bottom-up PC approach is comprised of a sequence of REML analyses that starts with a sub-set of traits. New traits/countries are added one by one into the analysis, and after each trait addition step the correct rank of the model is determined. The latter can be inferred based on the size of the smallest eigenvalues of **G **[5] or of the correlation matrix or by using likelihood based model selection tools such as Akaike's information criterion (AIC) [18], which takes into account both the magnitude of the likelihood and the number of parameters in the model, thus penalizing for overparameterized models. The latter was used in this study. For given starting values in each step, we decomposed **G **into **S **and **D**, estimated **D **conditional on **S **and combined **S **and **D **to update **G**. At the beginning of the analysis, starting values provided by Interbull were used and in the subsequent steps, estimates were obtained from the previous steps.

The rationale behind the bottom-up algorithm is to select in each step the highest rank, which is still justified by the AIC criteria. Each time a new country/trait, *k *+ 1, is added to the analysis, the variance of the previous traits is already completely described by the *r *eigenvectors. The genetic variance of the new trait and its covariance with the previous eigenvectors is estimated and if it is considered to provide new information on breeding values, the new breeding value equation and the new rank, *r *+ 1, is kept.

Implementation for MACE:

1. Initial step

(a) choose *k *countries as starting sub-set

(b) use starting values **G**_0_, take *EDC_ij _*and *λ_j _*for bull *i *to model the residual variance by applying weights *w_ij_*

(c) estimate *k *× *k *matrix  for the *k *starting countries under the full rank model, *r *= *k*

(d) calculate Akaike's information criterion value *AIC_r _*= 2 log *L *+ 2*p*, where log L is the maximum log Likelihood and *p *= *r*(*r *+ 1)/2 the number of parameters

2. Determination of the correct rank

(a) for a given rank decompose 

(b) derive , where  is obtained from  by removing the smallest eigenvalue from  and the corresponding eigenvector from 

(c) update the weights using , *EDC_ij _*and *λ_j_*

(d) estimate a new  with  and  as covariables by fitting model (5).

(e) calculate *AIC*_*r*-1_

(f) select the best model ("rank reduction" step)

• after the initial step: while *AIC*_*r*-1 _<*AIC*_*r*_, set *r *= *r*-1 and repeat step 2, otherwise take  and  and proceed to step 3

• after the country addition step: if *AIC*_*r*-1 _<*AIC_r_*, replace  and  with  and , otherwise take  and  and proceed to step 3

3. Addition of a new country/trait

(a) if *k < t*, *k *= *k *+ 1 and *r *= *r *+ 1

• add a new row and column of zeros to  and , and set the *k^th ^*element of  to 1 and the *r^th ^*diagonal element of  to twice the average genetic variance from countries *j *= 1, *k*. Two times the mean value was used as a starting value for estimation of the variance of a new country to improve the convergence of iteration.

(b) update the weights using , *EDC_ij _*and *λ_j _*(*w_ij _*= *EDC_ij_/g_jj_λ_j_*)

(c) estimate a new  and backtransform to  using Equation (5)

(d) calculate *AIC_r_*

4. repeat steps 2 and 3 until *k *= *t*

5. Final step: update the weigths and re-estimate the parameters

### Direct PC approach

Genetic principal components can be estimated directly from the data [13]. The genetic (co)variance matrix is decomposed into matrices of eigenvalues and eigenvectors and only the leading principal components with notable contribution to the total variance are selected to estimate the genetic parameters. The direct estimation method requires *a priori *knowledge of the number of principal components fitted in the model or it must be estimated.

#### Defining the correct rank of matrix

Meyer and Kirkpatrick [19] noticed that selecting too low a rank in the direct PC approach can lead to picking up the wrong subset of PC, which can result in biased estimates. Thus, it is important to select the correct rank when the direct PC approach is employed. We followed the procedure of Meyer and Kirkpatrick [19], to determine the appropriate rank and to test the capability of the bottom-up PC approach to define an appropriate rank. First, the (co)variance matrix for protein yield provided by Interbull was decomposed. Then we studied the magnitude of the eigenvalues to make an informed guess of the correct rank. After this, we performed several direct PC analyses with ranks bracketing this value. And finally, we examined the values of Log L and AIC, the sum of the eigenvalues, the magnitude of the leading eigenvalues to determine the correct rank. In addition, average quadratic deviations between *p *optimal and sub-optimal models, , were calculated to indicate changes in the estimates of genetic correlations while moving away from the optimal model [11].  was defined as(6)

where *t *is the number of traits and *r_ij,m _*is the estimated genetic correlation between traits *i *and *j *from an analysis fitting *m *PC. The genetic correlations from the sub-optimal models were contrasted with the estimates from the direct PC rank 20 model (*r_ij_*,_20_), which was the optimal rank selected by the bottom-up approach.

When the rank of the model is appropriately defined, [19] AIC should be at its minimum and the magnitude of the leading principal components and the sum of the eigenvalues stabilized, indicating that there is no re-partitioning of the genetic variance into the residual variance, which is the case if too few principal components are fitted [11]. Further, the improvement of the Log Likelihood beyond the optimal model is expected to be negligible.

### Differences between the direct and bottom-up PC approaches

The parameterization in the bottom-up PC approach differs from the direct PC approach in the matrix that is used for the eigenvalue decomposition. In the bottom-up PC approach, the eigenvalue decomposition was done on the correlation matrix, while in the direct PC approach the parameterization was on the (co)variance matrix [13]. For both PC approaches, the heterogeneity in residual variances were taken into account using weights, as outlined above. In the bottom-up PC approach, they were updated after each REML run, implying that  were fixed, whereas  were estimated in the direct PC approach.

### Test application

Data of the MACE Interbull Holstein protein yield and somatic cell count (SCC) evaluations were used for testing. Deregressed breeding values [20] for protein yield came from the August 2007 evaluation, consisting of 25 countries and those for SCC from the April 2009 evaluation comprising 23 countries. Table 1 lists the countries participating in the international evaluations in 2007 for protein yield and in 2009 for SCC. The number of countries differs between biological traits since some of countries - often those who joined the international evaluation only recently - provide data only for production traits. In addition, new countries join the MACE evaluation over time, so the number of countries involved increases gradually. We followed Interbull's practice by listing countries in all figures and tables (except Table 1 for SCC) based on their joining date for the evaluation of each biological trait.

**Table 1:** Structure of the datasets for protein yield and somatic cell count (SCC).

		Protein yield					SCC				
			
Country	Code	Number of bulls		Common bulls^a^			Number of bulls		Common bulls^a^		
					
		Total	Foreign bulls, % ^c^	Min^b^	Max^b^	Mean	Total	Foreign bulls^c ^, %	Min^b^	Max^b^	Mean
Canada	CAN	7028	33	2	1044	267	7730	34	4	1191	331
Germany	DEU	16734	23	56	1194	370	18624	25	49	1526	469
Dnk-Fin-Swe^d^	DFS	8900	13	12	590	248	9459	13	19	731	314
France	FRA	11127	20	3	568	220	12254	19	7	622	274
Italy	ITA	6322	20	8	607	253	7254	23	11	777	338
The Netherlands	NLD	9696	24	26	1194	346	10935	26	37	1526	481
USA	USA	23380	6	6	1044	410	25281	6	10	1191	507
Switzerland	CHE	715	37	4	209	118	946	45	9	325	182
Great Britain	GBR	4361	51	7	873	316	4017	55	12	855	377
New Zealand	NZL	4253	24	3	560	209	4886	22	6	725	255
Australia	AUS	4950	26	5	681	216	5404	31	12	895	325
Belgium	BEL	634	97	12	425	143	665	97	14	466	166
Ireland	IRL	1260	79	0	354	153	1337	96	3	388	183
Spain	ESP	1499	48	2	408	203	1720	45	3	455	246
Czech Republic	CZE	2036	75	12	590	202	2453	75	17	768	279
Slovenia	SVN	196	55	5	68	32	-^e^	-	-	-	-
Estonia	EST	472	46	2	93	30	556	49	6	117	40
Israel	ISR	773	11	0	59	27	853	11	1	68	33
Swiss Red Hol^f^	CHR	1162	45	3	256	103	1359	42	10	327	147
French Red Hol^f^	FRR	145	72	0	73	9	168	71	1	84	15
Hungary	HUN	1898	46	2	502	192	1638	63	5	573	246
Poland	POL	5071	16	0	295	118	-^e^	-	-	-	-
South Africa	ZAF	920	48	1	372	148	882	54	3	402	180
Japan	JPN	3177	67	1	226	97	3562	63	1	272	123
Latvia	LVA	232	71	6	71	29	-^e^	-	-	-	-
Danish Red Hol^f^	DNR	-^e^	-	-	-	-	232	38	1	83	16

Total number of bulls		116941					122215				

^a ^With other countries

^b ^Minimum (min) and maximum (max) values

^c ^Bull's country of first registration is embedded in its international identity and was extracted from it

^d ^Denmark, Finland and Sweden

^e ^Country does not participate in international evaluation for this trait

^f ^Holstein

The total number of records was 116 941 for protein yield and 122 215 for SCC. These represented 103 676 and 100 551 bulls with deregressed breeding values, respectively. The number of bulls with records in protein yield varied from 145 to 23 380 among countries, with a mean of 4 678 bulls per country.

Corresponding values for SCC were 168 to 25 281, with a mean of 5 314 bulls per country. For both biological traits, bulls were used mainly in one country; only 5% of the bulls were used in two countries and 1% in three countries. Further, only 286 bulls (i.e. 0.3%) with records for protein yield and 321 bulls (i.e. 0.3%) with records for SCC were used in more than 10 countries. Breeding policies vary notably among countries in terms of how much countries rely on their own breeding schemes or whether they import most of their breeding animals. USA is an example of a country that has a long tradition of Holstein breeding: only 6% of the bulls were imported bulls for the 2007 protein yield data (Table 1). Conversely, Belgium is an example of a country that leans heavily on import: in the same data, 97% of the Holstein bulls used in Belgium were imported (Table 1). The number of common bulls between countries varied from zero to 1 194 for protein yield, with a mean of 178, and for SCC from one to 1 526, with a mean of 240. Substantial variation existed in the number of common bulls among countries. For both biological traits, French Red Holstein shared the smallest number of common bulls with the other countries and the USA, as a popular trading partner, shared the most.

Bottom-up PC runs were performed for both traits. Direct PC runs with ranks 15, 17, 19, 20 and 25 were carried out for protein yield to evaluate the optimal rank using the methods proposed by Meyer and Kirkpatrick [19]. For SCC, however, only the rank suggested by the bottom-up PC approach was used in the direct PC analyses.

The sensitivity of the bottom-up PC approach to different orders of country addition was tested for a sub-set of nine countries: France, USA, Czech Republic, Latvia, Poland, New-Zealand, Australia, Slovenia and Ireland. These nine countries that were well and loosely linked, represented different hemispheres, and different managing systems and thus constituted a representative sample of all countries involved in the Interbull evaluation. Two different orders were tested. Order1 was the order of introduction of the countries above and order2 was the reverse of order1. For both orders, the analysis started with four countries.

The order of country addition should not affect the estimates, if only non-significant eigenvalues are excluded. To test this, we modified the bottom-up PC approach. Instead of selecting the best model based on the AIC (steps 2e-f, 3d), we determined a rank based on the proportion of explained variance in the transformation step 2a. Therefore, steps 2b-d became optional, depending on whether the rank was reduced or not. We tested three scenarios: the modified bottom-up approach was required to include 97, 99, or 99.5% of the total variance in the transformation step. For comparison, a full fit direct PC analysis (rank 9) and a basic bottom-up analysis were carried out for the sub-set of nine countries.

The WOMBAT software [21] was used for the direct PC analyses, as well as for the variance component estimation in the bottom-up PC approach. The average information REML algorithm was applied for both approaches. Bull pedigrees were based on sire and maternal grand sire information. Genetic correlations estimated by Interbull in their test runs (protein yield: test run preceding August 2007 evaluation, SCC: test run preceding April 2009 evaluation) were used for comparison.

## Results and Discussion

### Bottom-up approach - effect of the order of country addition on the results

Table 2 shows the effects of varying the order in which countries are added in the modified bottom-up PC approach on estimates of genetic correlations among the nine countries considered. Explaining 97, 99, and 99.5% of the total variance required the inclusion of the 6, 7 or 8 largest eigenvalues, respectively. Results clearly revealed the importance of the correct rank selection. When 99.5% of the variance in the eigenvalues was taken into account (rank 8), the order of the country addition had no influence on the estimates of the genetic correlations. Thus, relatively large number of PC were required to explain all necessary variation in the data. When a larger proportion of the variance in the eigenvalues was removed (ranks 7 and 6), the order of the countries added in the analysis affected the estimates of the genetic correlations. Especially the genetic correlations of Slovenia and Latvia with the other countries changed notably with the change in the order. Even though the variance explained by the 6th and 7th PC was small, those PC were, however, essential to be included in the analysis to ensure that all necessary PC were picked up. This phenomenon has also been observed in other studies [22,11]. The bottom-up PC approach and using AIC to determine the rank resulted in rank 8 as well, indicating that the algorithm was able to find the correct rank.

**Table 2:** The effect of the order of country addition on the estimates of the bottom-up PC approach for protein yield.

			Differences			
		
Countries^a^		Genetic correlations, direct PC 9	Direct PC 9 vs. Bottom-up PC rank 8	Bottom-up PC order1^b ^vs. order2^c^		
			
1	2			rank 8	rank 7	rank 6
FRA	USA	0.87	0	0	0	0.04
FRA	CZE	0.58	0	0	0	0.03
FRA	LVA	0.24	-0.02	0	0	0.24
FRA	POL	0.65	0	0	0	-0.02
FRA	NZL	0.68	0	0	0	-0.07
FRA	AUS	0.76	0	0	0	-0.01
FRA	SVN	0.51	-0.01	0.02	-0.14	-0.17
FRA	IRL	0.78	0	0	0.01	0
USA	CZE	0.59	0	0	0	0
USA	LVA	0.31	-0.01	0.01	0.02	-0.40
USA	POL	0.56	0	0	0	0.02
USA	NZL	0.54	0	0	0	-0.02
USA	AUS	0.65	0	0	0	0.05
USA	SVN	0.36	0.02	-0.03	-0.12	-0.08
USA	IRL	0.63	0	0	0.02	0.08
CZE	LVA	0.09	-0.04	0	0.03	-0.02
CZE	POL	0.55	0	0	0	-0.05
CZE	NZL	0.47	0	0.01	0.01	0
CZE	AUS	0.53	0	0	0	-0.06
CZE	SVN	0.44	0	0.04	0	-0.04
CZE	IRL	0.51	0.01	0	-0.02	-0.04
LVA	POL	0.62	-0.01	0	-0.01	-0.28
LVA	NZL	0.15	-0.05	0.02	-0.01	0.13
LVA	AUS	0.51	-0.03	0.01	-0.01	-0.08
LVA	SVN	0.21	0.07	-0.01	-0.12	0.16
LVA	IRL	0.33	0.02	0.02	-0.02	0.08
POL	NZL	0.49	0	0	0	0.06
POL	AUS	0.70	0	0	0	0.07
POL	SVN	0.57	0.01	0	-0.04	0.06
POL	IRL	0.68	0	0	0	0.04
NZL	AUS	0.80	0	0	0	0.01
NZL	SVN	0.34	-0.01	0.03	-0.14	-0.33
NZL	IRL	0.81	-0.01	0	0.01	-0.05
AUS	SVN	0.42	0.01	0.01	-0.14	-0.07
AUS	IRL	0.84	0	0	0.01	0.07
SVN	IRL	0.74	-0.03	0	-0.12	-0.13

Mean		0.54	-0.002	0.003	-0.021	-0.022
Mean_abs^d^		0.54	0.010	0.006	0.028	0.085
Max		0.87	0.07	0.04	0.14	0.40

For comparison, the estimates of the genetic correlations from the direct PC full rank model and the differences in the estimates of the genetic correlations from the direct PC full rank and the bottom-up PC rank 8 models are also presented. The mean and maximum (max) values of genetic correlations from the direct PC full fit and mean and max differences from above comparisons are shown at the bottom of the table.

^a ^Keys of the country codes are shown in Table 1

^b ^Order 1: FRA, USA, CZE, LVA, POL, NZL, AUS, SVN, IRL

^c ^Order 2 is reverse to order 1

^d ^Mean of the absolute differences

### Correct rank

Information used for the model selection of the protein yield data under the direct PC approach is summarized in Table 3. AIC for the 25-trait analysis was highest for a model fitting 19 PC and log likelihood did not increase significantly beyond rank 19. The sums of eigenvalues and the leading PC were, in practice, identical between models fitting ranks 19, 20 and 25. Furthermore, the last five eigenvalues equalled zero with a precision of two decimals, thus they included basically no information. Based on the  values, estimates of genetic correlations from the models fitting ranks 19, 20 and 25 were almost identical. Differences in the estimates started to increase, as the rank was dropped to 17 and 15. Thus, results suggested that either rank 19 or 20 is the appropriate rank to describe the genetic variation in protein yield. This means a reduction from 5 to 6% in the number of parameters needed to describe the complete 25 × 25 (co)variance matrix, because the number of parameters for the direct PC is *p *= *r*(2*t - r *+ 1)*/*2.

**Table 3:** Selection of the appropriate rank for protein yield under the direct PC approach.

	Rank 15	Rank 17	Rank 19	Rank 20	Full fit
	-68	-19	0	-4	-19
log L^b^	-105	-36	-2	0	0
	0.029	0.017	0.004	0	0.001
No of parameters	271	290	305	311	325
Sum of eigenvalues	1696	1695	1695	1695	1695
E1^d^	1326	1330	1331	1331	1331
E2	78.9	76.7	76.1	76.1	76.0
E3	69.8	65.0	60.3	60.1	60.1
E4	43.6	44.5	47.4	47.2	47.1
E5	36.6	35.2	33.2	33.0	33.1
E6	30.9	30.4	28.8	28.6	28.6
E7	22.3	21.3	21.4	21.3	21.3
E8	19.7	17.8	17.2	17.3	17.2
E9	15.0	15.4	16.2	15.9	16.0
E10	12.9	12.3	12.3	12.3	12.3
E11	10.6	10.5	10.6	10.6	10.6
E12	9.8	9.9	8.8	8.5	8.5
E13	9.2	8.6	8.4	8.3	8.3
E14	6.3	6.5	6.5	6.7	6.7
E15	4.3	5.2	5.2	5.2	5.2
E16		3.9	4.2	4.1	4.1
E17		2.7	3.2	3.3	3.3
E18			2.8	2.8	2.8
E19			1.1	1.3	1.3
E20				1.1	1.2
E21					0.0
E22					0.0
E23					0.0
E24					0.0
E25					0.0

^a ^Akaike's information criterion, expressed as deviation from highest value

^b ^Maximum Log Likelihood, expressed as deviation from highest value

^c ^A square root of the average squared deviation of the estimated genetic correlations. The estimates obtained under the direct PC rank 20 model were used as the estimates of comparison

^d ^Eigenvalues 1,...,25 of the **G **matrix

The bottom-up PC run terminated with rank 20 for protein yield, indicating that the approach is able to find the correct rank. Under the bottom-up PC, **G **is obtained by backtransforming it and only the matrix of eigenvalues is directly estimated, thus *p *= *r*(*r *+ 1)*/*2, and only 65% of the parameters were sufficient to describe the complete (co)variance matrix for that method. Based on the bottom-up results, the appropriate rank was 15 for SCC. Thus, only 44% of the parameters under the bottom-up PC were needed to describe the 23 × 23 (co)variance matrix for SCC, whereas the corresponding number for the direct PC rank 15 analysis was 87%.

Our results on the importance of fitting an optimal rank in the principal component analysis are supported by earlier studies by Meyer [22,11] and Meyer and Kirkpatrick [19]. While studying reduced rank multivariate animal models for beef cattle, Meyer noticed that fitting too few principal components resulted in inaccurate estimates of the genetic parameters [22,11]. A more recent study of Meyer and Kirkpatrick [19] has listed three sources of bias of reduced rank estimates: spread of sample roots, constraining estimates to the parameter space and picking up the wrong subset of the genetic PC, if too few PC are fitted.

### Comparison of genetic correlations

**Figure 1:**
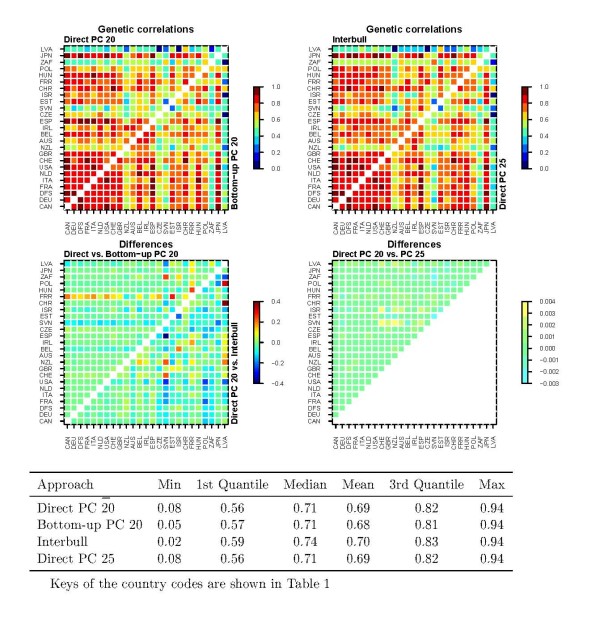
**Direct PC, bottom-up PC and Interbull estimates of genetic correlations for protein yield and differences in the estimates between the approaches**. Differences shown are estimates from the first method listed minus estimates from the second method.

**Figure 2:**
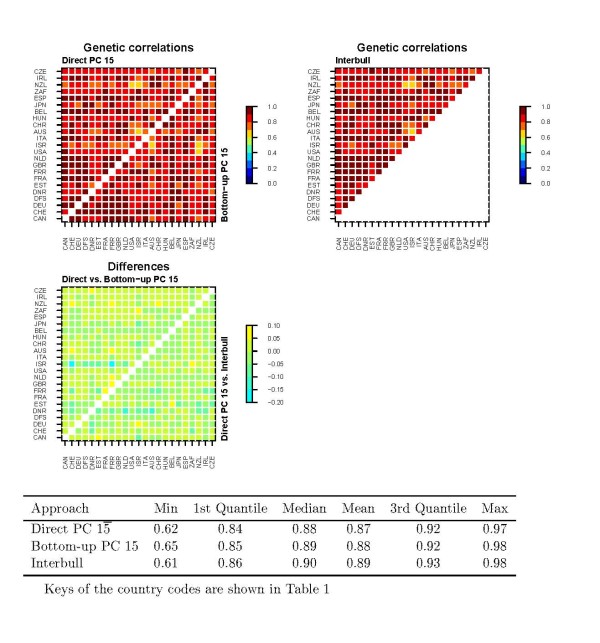
**Direct PC, bottom-up PC and Interbull estimates of genetic correlations for SCC and differences in the estimates between the approaches**. Differences shown are estimates from the first method listed minus estimates from the second method.

Figures 1 and 2 summarize the genetic correlations for protein yield and SCC, respectively. Heat map type plots demonstrate the magnitude of the genetic correlations among countries from different approaches, as well as the differences in genetic correlations between approaches. Descriptive statistics of the variation in the correlations from different approaches are collected in the tables below both figures. In general, differences in the estimates obtained with different approaches were small, especially for SCC. Genetic correlations for SCC were high in magnitude for all countries, whereas those for protein yield were very low for some countries - contrary to the biologically justified expectation of on average high genetic correlations. The different approaches did not vary in this respect.

The average estimates of genetic correlations from the direct PC rank 20, direct PC full fit, bottom-up PC rank 20 and Interbull analyses for protein yield were very similar, ranging from 0.68 to 0.70 (Figure 1). Based on the first and third quantiles and the median, the distribution of the Interbull estimates was on a somewhat higher level compared to those of the PC approaches. Nevertheless, the Interbull estimates included the lowest value for protein yield, being as low as 0.02 between New-Zealand and Latvia. The means of the SCC estimates were much higher, from 0.87 to 0.89 (Figure 2), compared to those for protein yield. In addition, the lowest values were rather high, ranging from 0.61 (Interbull) to 0.65 (bottom-up PC). The distributions of the estimates of genetic correlations from the different approaches were very similar for SCC, although those for the Interbull were on a slightly higher level. The plots of genetic correlations also showed that over-parameterization of the model for protein yield had virtually no effect on the estimates (Figure 1) since both rank 20 and 25 models resulted in almost identical genetic correlations.

**Figure 3:**
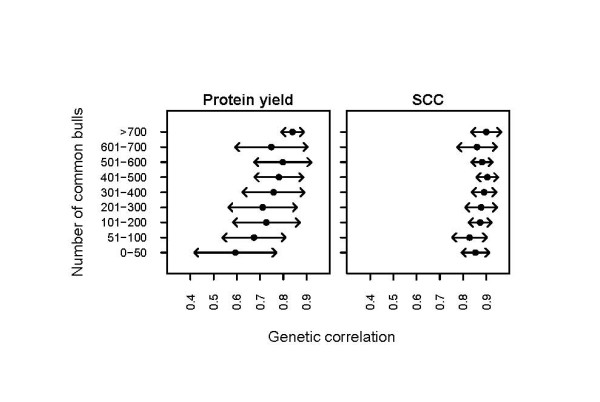
**Means ± one standard deviations of genetic correlations within classes of number of common bulls between countries**. The common bulls were defined as bulls having daughters in both countries of inspection without restriction on the country of origin of the bulls.

Figure 3 and Table 4 illustrate the challenges of the datasets used in this study. Plotting the genetic correlations with the number of common bulls between countries revealed that for protein yield, the level of the correlation estimates increased with the number of common bulls (Figure 3). This was, however, not the case for SCC. Furthermore, the standard deviations of the genetic correlations within classes defined by the number of common bulls were notably larger for protein yield than for SCC (Figure 3). In addition, a low number of common bulls was associated with larger differences in the estimates between the different approaches, hinting that the approaches reacted differently to challenges in the datasets.

**Table 4:** Dissection of the estimates of France, New-Zealand South-Africa, Slovenia and Latvia: magnitude of the genetic correlations and their standard errors for protein yield.

	FRA^a^	NZL	ZAF	SVN	LVA
	
	Genetic correlations				
Min	0.40	0.22	0.17	0.23	0.08
Median	0.80	0.56	0.49	0.51	0.43
Mean	0.76	0.57	0.49	0.50	0.40
Max	0.90	0.81	0.69	0.69	0.62

	**SEs of genetic correlations**				
	
Min	0.01	0.02	0.04	0.07	0.07
Median	0.02	0.03	0.05	0.08	0.08
Mean	0.03	0.05	0.06	0.09	0.09
Max	0.09	0.15	0.23	0.14	0.18

^a ^Keys of the country codes are shown in Table 1

On the one hand, the lack of connections between countries hinders the estimation of genetic parameters and this can explain the low genetic correlations for e.g. Slovenia and Latvia (Tables 1 and 4). On the other hand, countries like South-Africa and New-Zealand were also associated with a lower level of genetic correlations for protein yield (Table 4). However, on average, they have strong links with the other countries (Table 1). Furthermore, the standard errors of the estimates of New-Zealand and South-Africa with the other countries were relatively small, unlike those of Slovenia and Latvia with the other countries. Based on the study of Jakobsen et al. [1], different trait definitions and national genetic evaluation models, as well as genotype by environment interactions explain the low to moderate genetic correlations in international genetic evaluations of dairy bulls.

One of the main challenges in national genetic evaluation schemes incorporating foreign bulls is to adequately model systematic genetic differences by defining genetic groups. This holds especially for countries with small populations and where information on their daughters is scarce. This might result in proofs for foreign sires which are biased. As these national proofs are the data used in variance component estimation, inadequate genetic grouping at the national level may be one of the factors contributing to low estimates of genetic correlations for protein yield in different countries. Because selection of bulls is predominantly targeting production traits, the impact of ill-defined genetic groups on proofs for other, non-production traits is expected to be smaller. In addition, imported bulls may be more representative of the population in the country of origin as, for instance, for SCC.

Altogether, using a more parsimonious covariance structure did not resolve the problem of some small genetic correlations for protein yield. Currently, Interbull post-processes genetic correlations to correspond to the level of some justified expectation. This is done by utilizing information on the new estimates, estimates from the previous run, Interbull's own expectations and for non-Holstein breeds, correlations from Holstein [3]. Until the ultimate reason for the low estimates of genetic correlations has been identified, one alternative to the current post-processing would be to apply prior expectations under the Bayesian MACE suggested by, e.g., Mark et al. [23]. With insufficient data, the prior expectations would not be overridden and the level of the final estimates would be closer to their biologically justified expectations compared to the current non-post-processed estimates or those obtained under the PC approaches. By applying the approach suggested by Mark et al. [23], we might reduce the degree of parsimony which can be attained using the PC approaches, but this may be off-set by the prior information utilized and thus reduce mean square errors.

### Performance of the PC approaches

The run time of the direct PC analysis for protein yield reached a maximum for the rank 15 model (22 days), decreased with increasing rank, being shortest for the rank 20 model (5 days) and was 17 days for the full fit model. The memory needed for the direct PC rank 20 model for protein yield was 4.1 GB, whereas it was highest i.e. 6.3 GB for the full fit model. Thus, the costs of the over-parameterization were a longer run time and a higher RAM memory requirement without any increase in the accuracy of the estimation. Furthermore, the magnitude of standard errors of the estimates increased with the number of parameters to be estimated. This is a consequence of the increased sampling variance when estimating more parameters [see [22,11]]. Interestingly, fitting too few parameters in the model prolonged the run time. This occurred also for SCC (results not shown) and for the factor analytic models (manuscript in preparation). If the rank of the model is reduced too much, the number of available parameters is not sufficient to describe the (co)variance structure of the model, which in turn, detrimentally affects the convergence rate of the REML analysis.

The effects of the possible problems in the datasets accumulated as the bottom-up PC approach was used, which the protein yield data clearly demonstrated (Table 5). The first 15 countries introduced in the analysis were mostly well-linked countries that test many AI-bulls. They contributed 88% of the total data, but the computing time was less than 9% of the total time used. In the bottom-up approach, each time a new country/trait is added, the (co)variance matrix must be reestimated. Furthermore, the estimation process is carried out twice because two possible models are compared to test if the country addition requires an increase of rank or not. Thus, once difficulties in the iteration process have started, they will, at least to some extent, continue to the very end of the sequential country addition-rank reduction-process. On the other hand, when no larger problems are embedded in the data, the difference in the total estimation time between direct and bottom-up PC approaches is rather small, as demonstrated for SCC (Table 6).

**Table 5:** Run time (d:hr:min) and number of iterates required for analyses of protein yield.

	Country addition step			Rank reduction step			Total time
			
	Countries	Iterates	Time	Rank	Iterates	Time	
Bottom-up PC	7	5	0:00:46	7	4	0:00:26	0:01:12
	8	9	0:01:48	8	4	0:00:41	0:02:29
	9	8	0:02:21	9	5	0:01:12	0:03:33
	10	8	0:03:24	10	6	0:02:02	0:05:26
	11	11	0:06:05	11	5	0:02:25	0:08:30
	12	14	0:10:24	11	6	0:03:49	0:14:13
	13	13	0:10:53	12	6	0:03:50	0:14:43
	14	13	0:14:09	13	6	0:05:00	0:19:09
	15	12	0:16:34	14	5	0:05:28	0:22:02
	16	77	6:03:56	15	8	0:11:04	6:15:00
	17	12	1:06:04	16	6	0:10:40	1:16:44
	18	17	2:10:31	16	13	1:03:47	3:14:18
	19	12	1:13:49	17	6	0:13:00	2:02:49
	20	21	3:14:19	17	12	1:07:15	4:21:34
	21	14	1:22:37	18	5	0:13:08	2:11:45
	22	28	5:11:05	19	7	0:22:04	6:09:09
	23	15	3:14:23	19	11	1:15:25	5:04:48
	24	15	3:17:11	20	6	1:24:00	4:17:35
	25	14	4:05:09	20	12	2:04:03	6:09:12
							46:23:11
Direct PC	25			20	24	5:13:27	

**Table 6:** Run time (d:hr:min) and number of iterates required for analyses of SCC.

	Country addition step			Rank reduction step			Total time
			
	Countries	Iterates	Time	Rank	Iterates	Time	
Bottom-up PC	7	25	0:02:45	5	3+2+4^a^	0:00:28	0:03:13
	8	14	0:00:56	6	3	0:00:09	0:01:05
	9	13	0:01:29	7	5	0:00:22	0:01:51
	10	6	0:01:19	8	5	0:00:37	0:01:56
	11	6	0:01:58	9	5	0:00:56	0:02:54
	12	3	0:01:50	9	21	0:05:05	0:06:55
	13	11	0:03:59	10	5	0:01:21	0:05:20
	14	26	0:12:18	11	8	0:02:49	0:15:07
	15	22	0:13:43	11	6	0:03:11	0:16:54
	16	8	0:05:26	11	4	0:02:21	0:07:47
	17	9	0:06:01	11	4	0:02:17	0:08:18
	18	10	0:06:31	12	8	0:03:58	0:10:29
	19	12	0:10:09	12	6	0:04:04	0:14:13
	20	11	0:11:20	13	5	0:03:51	0:15:11
	21	13	0:14:34	14	7	0:06:25	0:20:59
	22	15	1:01:54	14	6	0:07:39	1:09:33
	23	9	0:13:13	15	7	0:07:59	0:21:12
							7:18:57
Direct PC	23			15	86	7:00:02	

^a ^Three rank reduction steps were needed before the appropriate rank was found.

Overall, both approaches tested in this study performed very well and estimates of genetic correlations were similar to the Interbull estimates. Both PC approaches were applied to complete datasets unlike those suggested in the earlier studies [7,12,9] and the current Interbull procedure [2,3]. One advantage of the direct over the bottom-up PC approach are the potentially much reduced computational requirements. This applies in particular when an analysis is started with a small subset of countries and countries with a problematic data structure are added in early on. Such problems were encountered for protein yield, resulting in the computational requirements shown in table 5. The current test version of the bottom-up PC approach has not been streamlined yet by any means. The analysis of the performance of the bottom-up approach (Tables 5 and 6) as well as preliminary tests give evidence that the computation time can be reduced by starting from a higher number of countries. Furthermore, when a new country is added, zero starting covariances between new and old countries could be replaced with covariances calculated from the mean correlation of countries already in the dataset and from the variances of those countries. The advantage of the bottom-up approach is that we estimate *r *elements of *r *× *r ***D***_r_*-matrix in the parameter estimation step and therefore, there is no danger of picking up the wrong subset of principal components.

## Conclusions

This study shows that both the direct and the bottom-up principal component approaches and the use of models with optimal rank are useful in the variance component estimation for MACE. Furthermore, both approaches can be applied to large datasets and data sub-setting is not needed. Based on the results, we emphasize the importance of the selection of the appropriate rank of the (co)variance matrix to obtain good estimates. The bottom-up PC approach is capable of determining the appropriate rank for highly over-parameterized models and thus leads to a more parsimonous variance structure. However, with a predetermined rank, the direct PC approach needs less computing time than the bottom-up PC. The third approach that is considered for variance component estimation for MACE is the direct factor analytic approach that will be presented in an upcoming paper.

## Competing interests

The authors declare that they have no competing interests.

## Authors' contributions

AMT performed the statistical analyses, modified the bottom-up PC approach and wrote the first draft of the manuscript. EAM developed the bottom-up PC approach and supervised the study. KM developed the direct PC approach, modified WOMBAT for the needs of this study and supervised the study. JJ and WFF provided the datasets. MHL, VD, WFF and JJ also supervised the study. All authors contributed to the writing of the manuscript.
